# Associations between emotion recognition and autistic and callous‐unemotional traits: differential effects of cueing to the eyes

**DOI:** 10.1111/jcpp.13736

**Published:** 2022-12-12

**Authors:** Virginia Carter Leno, Hannah Pickard, Liliana Cybulska, Tim Smith, Marcus Munafo, Ian Penton‐Voak, Emily Simonoff, Andrew Pickles, Rachael Bedford

**Affiliations:** ^1^ Institute of Psychology, Psychiatry and Neuroscience King's College London London UK; ^2^ Centre for Brain and Cognitive Development, Birkbeck University of London London UK; ^3^ School of Psychological Science University of Bristol Bristol UK; ^4^ MRC Integrative Epidemiology Unit University of Bristol Bristol UK; ^5^ National Institute for Health Research Biomedical Research Centre at the University Hospitals Bristol NHS Foundation Trust and the University of Bristol Bristol UK; ^6^ Department of Psychology University of Bath Bath UK

**Keywords:** Autism, callous‐unemotional, emotion recognition, eye gaze

## Abstract

**Background:**

Although autism and callous‐unemotional (CU) traits are distinct conditions, both are associated with difficulties in emotion recognition. However, it is unknown whether the emotion recognition difficulties characteristic of autism and CU traits are driven by comparable underpinning mechanisms.

**Methods:**

We tested whether cueing to the eyes improved emotion recognition in relation to autistic and CU traits in a heterogeneous sample of children enhanced for social, emotional and behavioural difficulties. Participants were 171 (*n* = 75 male) children aged 10–16 years with and without a diagnosis of autism (*n* = 99 autistic), who completed assessments of emotion recognition with and without cueing to the eyes. Parents completed the assessment of autistic and CU traits.

**Results:**

Associations between autistic and CU traits and emotion recognition accuracy were dependent upon gaze cueing. CU traits were associated with an overall decrease in emotion recognition in the uncued condition, but better fear recognition when cued to the eyes. Conversely, autistic traits were associated with decreased emotion recognition in the cued condition only, and no interactions between autistic traits and emotion were found.

**Conclusions:**

The differential effect of cueing to the eyes in autistic and CU traits suggests different mechanisms underpin emotion recognition abilities. Results suggest interventions designed to promote looking to the eyes may be beneficial for children with CU traits, but not for children with autistic characteristics. Future developmental studies of autism and CU characteristics are required to better understand how different pathways lead to overlapping socio‐cognitive profiles.

## Introduction

Recognising emotional facial expressions is important for understanding other's intentions and predicting behaviour, both critical components of everyday social interaction. Difficulties in emotion recognition are characteristic of both children diagnosed with an autism spectrum condition and with high autistic traits, and children with callous‐unemotional (CU) traits (characterised by low empathy, prosociality and sensitivity to others' emotions; Frick & White, 2008). However, it remains unclear if these apparently overlapping difficulties in emotion recognition are due to different underlying mechanisms. Defining the transdiagnostic factors that contribute to socio‐affective difficulties is important in terms of building models to understand typical social behaviour but also for developing interventions that target the underlying mechanism of atypicality.

Individuals with high levels of autistic traits and those with a clinical diagnosis of autism demonstrate emotion recognition difficulties, particularly for emotions that are more difficult to recognise (i.e., surprise, fear, anger; Losh et al., 2009; Lozier, Vanmeter, & Marsh, 2014), although reviews of the field note substantial heterogeneity of effects, which is likely driven by both sample and experiment‐related factors (Harms, Martin, & Wallace, 2010). Similarly, studies report that high CU traits are associated with impairments in the recognition of negative emotions such as fear and sadness (Marsh & Blair, 2008; Martin‐Key, Graf, Adams, & Fairchild, 2018), although meta‐analyses suggest that effects are found across both positive and negative emotions (see Dawel, O'Kearney, McKone, & Palermo, 2012 for a review).

Differential patterns of attention to facial features, specifically a lower inclination to look towards the eyes, have been suggested to underlie difficulties in emotion recognition. The eyes are a site of key information about the emotional valence of a face; they are the most viewed region during emotion recognition tasks, especially when viewing faces with negative facial expressions (Scheller, Büchel, & Gamer, 2012). Additionally, a case study of a patient with amygdala damage (patient SM), who had impairments recognising fear, found recognition difficulties were no longer present when the patient was instructed to look at the eyes (Adolphs et al., 2005). In autism, eye‐tracking studies suggest that autistic youth use different strategies to determine the emotional expressions of faces, with less time spent looking at the eyes (Klin, Jones, Schultz, Volkmar, & Cohen, 2002), and more time looking at the mouth (Spezio, Adolphs, Hurley, & Piven, 2007), although there is some evidence these differences may only be present in dynamic scenes (Speer, Cook, McMahon, & Clark, 2007) and are age‐dependent (Black et al., 2017). However, removing information from the eyes in emotion recognition tasks (either by freezing or bisecting the eye area) reduces performance in autistic children, suggesting that autistic youth are relying on the eye region to some extent (Back, Ropar, & Mitchell, 2007; Leung, Ordqvist, Falkmer, Parsons, & Falkmer, 2013). There is evidence that CU traits are associated with less looking to the eyes when viewing faces (Dadds, El Masry, Wimalaweera, & Guastella, 2008; Martin‐Key et al., 2018). CU traits are negatively correlated with spontaneous eye contact with mothers during parent–child interaction in childhood (Dadds et al., 2014), and one study found emotion recognition performance improves in boys with CU traits when explicitly cued to pay attention to the eyes (Dadds et al., 2008).

A growing literature has focused on comparing emotion recognition difficulties associated with autism and CU traits. Schwenck et al. (2012) compared boys with autism, conduct disorder with CU traits, conduct disorder without CU traits and a typically developing group and found no differences in emotion recognition accuracy. Bedford et al. (2020) reported that associations between CU traits and emotion recognition difficulties in typically developing children became non‐significant when adjusting for autistic traits, suggesting a potential commonality in emotion processing. However, two studies of autistic adolescents both found that CU traits were associated with poorer fear recognition (Carter Leno et al., 2015, 2020), indicating that CU traits may be associated with additional emotion recognition difficulties above and beyond those accounted for by autism. In terms of commonalities in processing styles that could underpin decreased behavioural performance, Bours et al. (2018) found that both autism diagnosis and CU traits were associated with reduced attention to the eyes when viewing fearful expressions (although the autism effect appeared to be non‐specific, with decreased looking found across a range of emotional expressions), and one study of autistic participants found that CU traits were associated with less looking to the eye area (Carter Leno et al., 2020). Thus, it remains unclear from existing comparative studies whether (a) autism and CU traits are associated with similar emotion recognition difficulties, and (b) if there are commonalities, whether these are driven by similar or different cognitive mechanisms.

In the current study, we tested emotion recognition ability in a heterogeneous sample of children who were enriched for social, emotional and behavioural difficulties. We directly manipulated attention to the eyes by comparing performance in cued (to the eyes) versus uncued conditions. Based on our pre‐registered analyses (https://osf.io/p8yn9/), we hypothesised that both autistic and CU traits would be associated with lower emotion recognition accuracy, and primarily be driven by impairments in the recognition of fear for CU traits, but driven by emotions that are more difficult to identify (e.g. surprise, anger, fear) for autistic traits. We sought to replicate the effect reported by Dadds et al. (2008), such that cueing to the eyes improves emotion recognition performance in children with CU traits, and this is driven by an improvement in the recognition of fear. We did not pre‐register any hypotheses regarding the effect of cueing to the eyes on autism‐related emotion recognition difficulties due to the heterogeneity of existing literature.

## Methods

### Participants and study design

Participants were recruited via secondary schools, charities and social media. An additional targeted recruitment drive aimed at increasing variability in autistic and CU traits in the sample recruited through schools specifically for children with social, emotional and behavioural difficulties. Inclusion criteria were being 10–16 years of age, living in the UK and being fluent in English. Exclusion criteria were the child having parent‐reported genetic or psychotic conditions. Parent‐rated questionnaires and child‐completed tasks were presented online using Qualtrics and Gorilla, respectively, as part of a wider project (see Appendix S1 and Table S1 for details). Autism diagnosis was recorded using parent‐reported diagnostic information (which diagnostic label, who gave the diagnosis, and age of diagnosis). Where two siblings took part (*n* = 2), data from one sibling were excluded at random. Full sample characteristics are reported in Table 1. To be included in the current analysis, participants had to have complete data on either condition in the emotion recognition task, parent‐reported autistic and CU traits, parental education (a proxy for socio‐economic status), home environment and verbal IQ (VIQ). A total of 204 participants completed the emotion recognition task, 203 participants had valid emotion recognition task data and 171 participants had valid emotion recognition data and valid covariate data (see Appendix S2 for details of cognitive task data processing and exclusion criteria). We tested for group differences in age, sex, ethnicity (coded as White vs. Non‐White for statistical analysis), parental education, VIQ, CHAOS total, CU traits and autistic traits between the final analysis sample who had valid data on the emotion recognition task + all model covariates (*n* = 171) and the subsample who had complete data on the first part of the emotion recognition task only and were therefore excluded from the analysis (*n* = 33). There was some evidence of group differences in parental education (two‐sided Fisher's exact test, *p* = .05), such that the excluded sample had higher levels of parental education (97%) than the included sample (83%), and age (*t*(202) = −2.01, *p* = .05), such that the excluded sample was younger. All other comparisons were non‐significant (*p*s > .19). All parents provided consent, and children provided assent. This study was approved by the Psychiatry, Nursing and Midwifery Research Ethics Committee, King's College London (HR‐19/20‐17193) and Bath Ethics Committee (Psychology Research Ethics Committee reference number 20‐199).

**Table 1:** Sample descriptives for included participants (*N* = 171)

	Mean (*SD*; range)
Male:female (% female)	75:96 (56%)
Child age (years)	13.14 (1.76; 10.03–16.88)
Household CHAOS score	4.33 (3.74; 0–14)
Parental education	
<UG degree: ≥UG degree (% ≥ UG degree)	32:139 (81%)
Ethnicity	
White English/Welsh/Scottish/Northern Irish/British	132 (77%)
Other White Background	15 (9%)
Other Mixed/Multiple Background	6 (4%)
Indian	3 (2%)
Other Ethnic Group	3 (2%)
African	2 (1%)
Other Black/African/Caribbean/Black British Background	2 (1%)
Other categories with <*n* = 2 participants including Prefer Not to Say	8 (5%)
VIQ *t* score	119.86 (18.93; 55–145)
ICU total	25.20 (12.01; 3–63)
Above ICU cut‐off (%)	48 (28%)
SRS‐Brief	17.29 (12.51; 0–42)
SCQ‐Lifetime	12.74 (9.03; 0–31)

CHAOS, Confusion, Hubbub and Order Scale; ICU, Inventory of Callous‐Unemotional Traits; SCQ, Social Communication Questionnaire; SRS, Social Responsiveness Scale; UG, undergraduate; VIQ, verbal IQ.

### Measures

#### Parent‐report measures

The Inventory of Callous‐Unemotional Traits (ICU; Frick, 2004) was used to measure CU traits. The ICU includes 24 items that tap the affective features of CU traits, with higher scores associated with higher levels of conduct problems and psychosocial impairment (Essau, Sasagawa, & Frick, 2006). Clinical cut‐offs have been proposed for youth aged 11–16 using sex‐based norms at the 90th percentile (Kemp et al., 2021); we report a percentage above the cut‐off to aid sample characterisation. The ICU showed good internal consistency in the current sample (α = .90).

The Social Responsiveness Scale‐Brief was used to measure autistic traits (Moul, Cauchi, Hawes, Brennan, & Dadds, 2015). The SRS‐Brief is based on an alternative scoring of the SRS (Constantino & Gruber, 2012), which consists of 65 statements about their child's behaviour over the last 6 months. The SRS‐Brief has been suggested as a measure of autistic traits which is less influenced by co‐occurring emotional and behavioural problems and has equivalent sensitivity (0.96) and a lower false positive rate than the original SRS scoring (0.58 as opposed to 0.75; Moul et al., 2015). The SRS‐Brief showed excellent internal consistency in the current sample (α = .96).

The Confusion, Hubbub and Order Scale (CHAOS; Matheny, Wachs, Ludwig, & Phillips, 1995) was used to assess and account for variations in the child's home environment that could impact task performance. The CHAOS is thought to measure aspects of the home environment (e.g. disorganisation), which are distinct from socio‐demographic measures, and showed good internal consistency in the current sample (α = .85).

#### Cognitive measures

Emotion recognition task: In this task, there were two conditions to enable us to measure the effect of cueing to different aspects of the face on emotion recognition ability. First, we measured uncued emotion recognition ability. Participants were shown a block of 20 ‘uncued’ trials consisting of faces expressing five emotions (happiness, sadness, surprise, anger and fear), with four trials per emotion. Four different face prototypes were used, taken from Griffiths et al. (2019), including two European faces (one male and one female) and two South Asian faces (one male and one female). An intensity level of 6 (from a possible range of 0–8) was selected to ensure sufficient variability in response accuracy (based on the location of maximal group differences in Griffiths et al., 2019). Each trial consisted of a central fixation cross on the screen for 1,000 ms, followed by the face stimulus for 2,000 ms, followed by five emotion labels.

To measure cued emotion recognition ability, participants were shown 60 trials using the same face stimuli. This version had three separate conditions (a) no cue condition – no fixation cross presented, (b) eye cue condition – fixation cross presented in between the eyes, and (c) nose cue condition – fixation cross presented on the tip of the nose, with 20 trials per condition. The trial condition was randomised, and stimuli were presented with equal probability to the left and right side of the screen to prevent anticipatory looking or practice effects. In the no cue condition, each trial consisted of a blank white screen for 1,000 ms, followed by the face stimulus on a white background for 2,000 ms, followed by five emotion labels. In the eye and nose cue conditions, each trial consisted of a fixation cross on the nose or in between the eyes, for 1,000 ms, followed by the face stimulus for 2,000 ms, followed by five emotion labels. For the current study, data from the eye cue condition only (20 trials) were compared with the initial uncued block at the beginning (20 trials). This was to ensure that the exposure to trials which cued participants to the eyes did not bias performance in uncued trials. For both the uncued and cued versions of the task (henceforth referred to as the uncued and cued condition), the position of the emotion labels was randomly chosen for each participant but remained consistent throughout. Participants were instructed to use the cursor to select the correct emotion label. The labels remained on the screen until the participant had made a response. Percentage accuracy was calculated as the sum of correct responses/total number of valid trials*100 for each emotion separately. Following trial and task‐level exclusions, one participant was excluded, and the remaining participants had an average of 97% valid trials.

Receptive one word picture vocabulary test‐4th edition (ROWPVT‐4): The ROWPVT is a vocabulary test designed for individuals from 2 to 80+ years adapted for online use following approval from the publishers. On each trial, the participant was presented with four pictures and an audio clip of a word that matched one of the pictures. Participants were instructed to select the picture that matched the word and could replay the audio clip as many times as required. VIQ was calculated by summing correct responses and converting the raw total to standardised *t* scores. Following trial and task‐level exclusions, two participants were excluded.

### Statistical analysis

All data processing and analysis were conducted in R, Stata 16 and SPSS 27. Analyses were pre‐registered on the Open Science Framework (https://osf.io/p8yn9/). See Supplementary Materials for details on how we deviated from our original pre‐registered protocol. To test the effect of cueing on the eyes on performance, we ran a generalised estimating equation (GEE) using an ordinal model with a logit link function, an unstructured working correlation matrix. When models did not converge, an exchangeable or independent correlation matrix was used instead. All models were run with robust standard errors to ensure results were robust to potential misspecification of the covariance matrix. Models included the main effect of gaze cue (no cue vs. eye cue), the main effect of emotion (fear, anger, happiness, sadness and surprise), a main effect of autistic traits, the main effect of CU traits, two‐way interactions between autistic traits*gaze cue, CU traits*gaze cue, autistic traits*emotion and CU traits*emotion, and two three‐way interactions between autistic traits*emotion*gaze cue and CU traits*emotion*gaze cue. Child sex, age, highest parental education (coded as no undergraduate degree/undergraduate degree or higher), household CHAOS score and VIQ scores were included as covariates. Our measures of autistic traits and CU traits were correlated at *r* = .56, *p* < .001 with a VIF of 1.50, suggesting it was acceptable to include both predictors in the same model. As we entered both autistic traits and CU traits as predictors, reported effects are interpreted as the coefficient for one domain adjusting for the effect of the other (and vice versa).

#### Hypotheses

*Hypothesis 1a*: Both autistic and CU traits will be associated with lower emotion recognition accuracy, as indicated by a significant main effect of CU traits and autistic traits.

*Hypothesis 1b*: There will be specificity in the nature of associations between CU/autistic traits and emotion recognition, such that impairments associated with CU traits are driven by difficulties in the recognition of fear, whereas impairments associated with autistic traits are driven by emotions that are more difficult to identify (e.g. surprise, anger, fear). These hypotheses would be supported by significant CU traits*emotion and autistic traits*emotion interaction terms in the uncued condition.

*Hypothesis 2*: Cueing to the eyes will improve emotion recognition performance in children with CU traits, and this will specifically be driven by an improvement in the recognition of fear. This hypothesis would be supported by a significant CU*gaze cue*emotion interaction and also by our pre‐specified CU*gaze cue interaction for fear recognition only.

#### Exploratory analyses

We tested whether the effect of cueing was dependent upon the level of autistic traits and whether this was specific to particular emotions, by including autistic traits*gaze condition and autistic traits*gaze condition*emotion interactions. We did not have any specific hypotheses here due to the heterogeneity of existing literature.

#### Supplementary analyses

To investigate the specificity of effects on CU traits, we conducted an additional analysis, which was not part of our pre‐registered protocol, where we re‐ran models adjusting for conduct problems (as measured by the Strengths and Difficulties Questionnaire conduct problems subscale; Goodman, Ford, Simmons, Gatward, & Meltzer, 2000). For brevity, we only report effects for conduct problems, autistic traits and CU traits in these adjusted models.

For all analyses, unstandardized estimates (*B*) and 95% confidence intervals (CIs) are presented for continuous and binary predictors to aid the interpretation of directionality (standardised estimates are not available for ordinal predictors or interaction terms in SPSS). As outcomes are ordinal these are log‐odds.

## Results

Emotion recognition accuracy across emotions and conditions is shown in Table 2. For full transparency and to aid reproducibility, we also provide task performance tabulated by CU traits and autism diagnosis (Table S2).

**Table 2:** Descriptive statistics for emotion recognition task

Emotion	Mean accuracy (*SD*; range)	
	Uncued condition (*n* = 171)	Cued condition (*n* = 167)
Anger	84.21 (22.18; 0–100)	90.12 (21.07; 0–100)
Happiness	87.72 (19.05; 0–100)	90.42 (18.15; 0–100)
Sadness	88.16 (17.85; 25–100)	90.72 (17.02; 0–100)
Fear	52.78 (28.11; 0–100)	58.53 (30.90; 0–100)
Surprise	83.77 (20.38; 25–100)	84.28 (21.34; 25–100)
Overall	79.33 (12.14; 40–100)	82.81 (13.16; 40–100)

### Omnibus model

An ordinal logistic GEE with an unstructured correlation matrix showed a main effect of emotion (*p* < .001) and gaze cue (*B* = −.40, 95% CIs [−.588, −.201], *p* < .001; see Table 3), such that accuracy was higher in the cued versus uncued condition. Follow‐up contrasts using the Wilcoxon‐Signed Rank Test for non‐parametric data indicated accuracy for fear was significantly lower than all other emotions (all *p*s < .001) and that accuracy for surprise was lower than accuracy for anger (*p* = .043), sadness (*p* < .001) and happiness (*p* < .001). There was no emotion*gaze cue interaction (*p* = .108). There was a significant effect of VIQ (*B* = .01, 95% CIs [.002, .018], *p* = .015), such that higher VIQ was associated with higher accuracy, a significant autistic traits*gaze cue interaction (*p* = .034), and, in support of Hypothesis 2, a significant CU traits*emotion*gaze cue interaction (*p* = .019). We followed up the significant interaction terms by running ordinal GEE models for uncued and cued conditions separately.

**Table 3:** Emotion recognition omnibus model results

	Wald χ^2^	Log‐Odds	95% CIs	*p* value
Child sex^a^	3.435	−.267	[−.550, .015]	.064
Child age	.933	.041	[−.042, .123]	.334
Household CHAOS score	.336	−.013	[−.057, .031]	.562
Parental education^b^	3.324	.328	[−.025, .681]	.068
VIQ	5.912	.010	[.002, .018]	**.015**
CU traits	.230	−.003	[−.017, .010]	.632
Autistic traits	3.564	−.014	[−.028, .001]	.059
Emotion	423.609			**<.001**
Condition	16.002	−.395	[−.588, −.201]	**<.001**
Condition*Emotion	7.596			.108
CU traits*Emotion	4.470			.346
CU traits*Condition	2.308			.129
Autistic traits*Emotion	1.975			.740
Autistic traits*Condition	4.472			**.034**
CU traits*Emotion*Condition	11.835			**.019**
Autistic traits*Emotion*Condition	1.989			.738

CHAOS, Confusion, Hubbub and Order Scale; VIQ, verbal IQ.

*p* values were bolded if *p* < .05.

^a^ Coefficient gives the effect on male compared to female.

^b^ Coefficient gives the effect of no undergraduate degree compared to ≥undergraduate degree.

### Uncued condition

**Figure 1:**
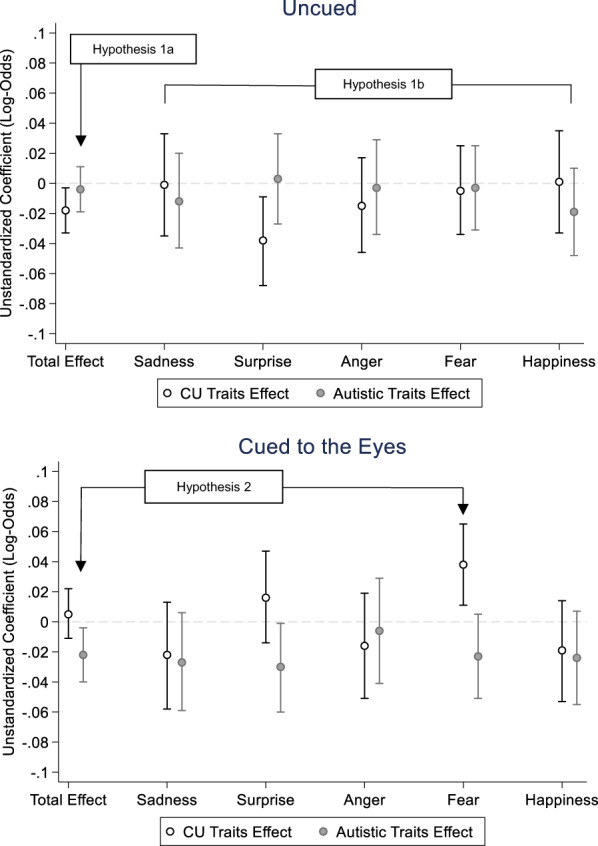
Associations between autistic and callous‐unemotional (CU) traits and emotion recognition accuracy in uncued and cued conditions. The dashed line indicates a coefficient of 0, that is no effect. We did not find any significant interaction effects with emotion in the uncued condition but were present for completeness

In the uncued condition (see Table 4), a GEE model with an unstructured correlation matrix showed a main effect of emotion (*p* < .001), with follow‐up contrasts indicating accuracy, was lower for fear than all other emotions (all *p*s < .001), and accuracy for surprise was lower than accuracy for sadness (*p* = .038) and happiness (*p* = .043). Results showed the main effect of parental education (*B* = .44, 95% CIs [.070, .819], *p* = .020), such that participants with parents with no undergraduate degree had higher accuracy compared to those with an undergraduate degree or higher, and the main effect of sex (*B* = −.30, 95% CIs [−.605, .004], *p* = .053), such that females had higher accuracy than males. In support of Hypothesis 1a, results also showed a main effect of CU traits (*B* = −.02, 95% CIs [−.033, −.003], *p* = .021), such that having higher CU traits was associated with lower accuracy (see Figure 1). Contrary to Hypothesis 1a, we did not find a significant effect of autistic traits (*B* = .01, 95% CIs [−.019, .011], *p* = .631). Both the CU traits*emotion and the autistic traits*emotion interaction were non‐significant (*p*s > .290).

**Table 4:** Emotion recognition model results, uncued condition

Uncued accuracy	Wald χ^2^	Log‐Odds	95% CIs	*p* value
Child sex^a^	3.746	−.301	[−.605, .004]	**.053**
Child age	.825	.045	[−.052, .141]	.364
Household CHAOS score	.016	−.003	[−.047, .041]	.900
Parental education^b^	5.414	.444	[.070, .819]	**.020**
VIQ	1.923	.006	[−.003, .015]	.166
CU traits	5.350	−.018	[−.033, −.003]	**.021**
Autistic traits	.231	−.004	[−.019, .011]	.631
Emotion	294.592			**<.001**
CU traits*Emotion	4.973			.290
Autistic traits*Emotion	2.452			.653

CHAOS, Confusion, Hubbub and Order Scale; VIQ, verbal IQ.

*p* values were bolded if *p* < .05.

^a^ Coefficient gives the effect on male compared to female.

^b^ Coefficient gives the effect of no undergraduate degree compared to ≥undergraduate degree.

### Cued condition

In the cued condition (see Table 5), a GEE model with an independent correlation matrix showed a main effect of emotion (*p* < .001), VIQ (*B* = .02, 95% CIs [.005, .026], *p* = .005), with follow‐up contrasts indicating accuracy was lower for fear than all other emotions (all *p*s < .001), and accuracy for surprise was lower than accuracy for anger (*p* = .004), sadness (*p* = .002) and happiness (*p* = .003). Results also showed the main effect of autistic traits (*B* = −.02, 95% CIs [−.040, −.004], *p* = .017), such that higher autistic traits were associated with lower accuracy. There was no significant main effect of CU traits (*B* = .01, 95% CIs [−.011, .022], *p* = .538). In support of Hypothesis 2, results showed a significant CU trait*emotion interaction (*p* = .010). When the cued performance was split by emotion, CU traits were associated with better accuracy for fear (*B* = .04, 95% CIs [.011, .065], *p* = .005). No associations were found with accuracy for any other emotion (*p*s > .218; see Figure 1). Our CU findings were further supported by our pre‐specified GEE for fear only, which showed significant CU traits*gaze cue interaction (*p* = .006).

**Table 5:** Emotion recognition model results, cued condition

Cued accuracy	Wald χ^2^	Log‐Odds	95% CIs	*p* value
Child sex^a^	1.491	−.240	[−.624, .145]	.222
Child age	2.316	.082	[−.024, .188]	.128
Household CHAOS score	.277	−.015	[−.072, .042]	.599
Parental education^b^	.653	.215	[−.306, .736]	.419
VIQ	8.026	.016	[.005, .026]	**.005**
CU traits	.380	.005	[−.011, .022]	.538
Autistic traits	5.661	−.022	[−.040, −.004]	**.017**
Emotion	191.756			**<.001**
CU traits*Emotion	13.192			**.010**
Autistic traits*Emotion	1.187			.880

CHAOS, Confusion, Hubbub and Order Scale; VIQ, verbal IQ.

*p* values were bolded if *p* < .05.

^a^ Coefficient gives the effect on male compared to female.

^b^ Coefficient gives the effect of no undergraduate degree compared to ≥undergraduate degree.

### Supplementary analyses including conduct problems as a predictor

#### Omnibus model

Results showed a main effect of conduct problems (*B* = −.14, 95% CIs [−.230, −.051], *p* = .002), such that participants with higher conduct problems had lower emotion recognition. The main effects of autistic traits and CU traits remained non‐significant (*p*s > .269). The two‐way autistic traits*gaze cue interaction was no longer statistically significant (*p* = .088), the two‐way CU traits*gaze cue interaction was now statistically significant (*p* = .038) and the three‐way CU traits*emotion*gaze cue interaction (*p* = .005) remained significant, suggesting that even when adjusting for conduct problems, the association between CU traits and emotion recognition was dependent upon both cueing and emotion. The three‐way autistic traits*emotion*gaze cue interaction remained non‐significant (*p* = .812).

#### Uncued condition

There was a significant main effect of conduct problems (*B* = −.11, 95% CIs [−.220, −.001], *p* = .049) and an emotion*conduct problems interaction (*p* = .024). When the performance was split by emotion, conduct problems were associated with lower accuracy for sadness (*B* = −.36, 95% CIs [−.565, −.163], *p* < .001). No associations were found with accuracy for any other emotion (*p*s < .39). The main effect of CU traits in the uncued condition was no longer significant (*B* = −.01, 95% CIs [−.021, .012], *p* = .580), suggesting that when adjusting for conduct problems, the association between CU traits and uncued emotion recognition was no longer present. The main effect of autistic traits remained non‐significant (*B* = .01, 95% CIs [−.016, .017], *p* = .969). Two‐way interactions between CU traits*emotion and autistic traits*emotion both remained non‐significant (*p*s > .102).

#### Cued condition

There was a main effect of conduct problems (*B* = −.19, 95% CIs [−.295, −.080], *p* < .001), but the two‐way conduct problems*emotion interaction was not significant (*p* = .935). The main effect of autistic traits was no longer significant (*B* = −.02, 95% CIs [−.034, .003], *p* = .102), suggesting that when adjusting for conduct problems, the association between autistic traits and cued emotion recognition was no longer present. The two‐way autistic traits*emotion remained non‐significant (*p* = .896). The main effect of CU traits remained non‐significant (*B* = .02, 95% CIs [−.002, .033], *p* = .089). The two‐way CU traits*emotion interaction remained significant (*p* = .030), which, as in primary models, was driven by a positive association between CU traits and fear recognition only (*B* = .05, 95% CIs [.019, .074], *p* = .001), suggesting that even when adjusting for conduct problems, cueing to the eyes led to a significant improvement in fear recognition in participants with CU traits, such that those with higher traits were now more accurate at recognising fear.

## Discussion

Current results suggest a dissociation in the underlying mechanisms that drive emotion recognition difficulties in children with autistic versus CU traits. CU traits were associated with reduced emotion recognition accuracy within the uncued condition, but *better* fear recognition in the cued condition. Autistic traits showed the reverse effect, such that they were only associated with reduced emotion recognition accuracy in the cued condition.

Results showed a significant interaction between CU traits, gaze cues and emotion. When this was broken down, we found the main effect of CU traits in the uncued condition, such that higher CU traits were associated with lower emotion recognition accuracy, in line with meta‐analyses reporting pervasive emotion recognition impairments in individuals with CU traits (Dawel et al., 2012). Unlike others (e.g. Marsh & Blair, 2008; Martin‐Key et al., 2018), we did not find a specific impairment in fear recognition, rather participants with higher levels of CU traits performed worse across all emotion types in the uncued condition. However, we highlight that in our supplementary analyses, where we adjusted for conduct problems, the effect of CU traits in the uncued condition fell below significance (in line with previous studies; Kohls et al., 2020). As these analyses were not part of our pre‐registered protocol, we interpret them with caution, but they do emphasise the importance of considering co‐occurring psychiatric traits when investigating the aetiological relevance of cognitive profiles. In the cued condition, results showed a CU traits*emotion interaction, driven by better recognition of fear. We consider the current result that cueing to the eyes improved emotion recognition accuracy in adolescents with CU traits, independent replication of work by Dadds et al. (2008). However, our work extends previous results to a larger and more heterogenous population of children; the original study sample consisted of middle‐ to high‐SES boys only. In terms of the positive association between CU traits and fear recognition when cued to the eyes, although there are many reports of poorer fear recognition in individuals with CU characteristics (Marsh & Blair, 2008; White et al., 2016), some have also reported opposite effects (Martin‐Key et al., 2018; Woodworth & Waschbusch, 2008). Additionally, most work that finds negative associations did not cue participants to look into the eyes – based on the current findings this may be an important modifier of the directionality of associations between CU traits and facial emotion recognition task performance. Previous work in typically developing 7 years old also found a trend between higher CU traits and quicker recognition of fear in a dynamic emotion recognition paradigm when adjusting for autistic traits (Bedford et al., 2020). One interpretation is that CU traits are associated with a lower inclination to prioritise attention towards certain areas of the face (or social stimuli in general), but when attention is drawn to the eyes (either by cueing participants with a fixation cross as in the current study, or using dynamic stimuli), this ameliorates emotion recognition difficulties, and can even reveal cognitive strengths.

Results also showed a significant autistic traits*gaze cue interaction, driven by a negative association between autistic traits and task performance when cued to the eyes. This pattern of effects is consistent with evidence that children with autistic traits use different strategies to process facial expressions (Harms et al., 2010; Joseph & Tanaka, 2003). In the uncued condition, participants with autistic traits appear unimpaired, potentially through compensatory strategies that allow them to reach the correct answer through an alternative route. However, when cued to the eyes, they may have difficulty because they are prevented from using their preferred strategy (e.g. relying more on information from the mouth area). This is not to say autistic children do not make use of any information from the eye region in emotion recognition tasks – paradigms that reduce the information available from the eye region find this leads to a reduction in performance in autistic children (Back et al., 2007; Leung et al., 2013), suggesting autistic youth must be gathering some information from the eye region when decoding emotional expressions. However, current results suggest that encouraging children with high levels of autistic traits to look at the eyes is not sufficient to ameliorate emotion recognition difficulties, potentially either because the eyes are less informative due to atypical processing of gaze‐related information, as suggested by imaging studies (e.g. Davies, Dapretto, Sigman, Sepeta, & Bookheimer, 2011), or because direct eye contact increases arousal and consequently interferes with autistic people's ability to process relevant facial cues (e.g. the eye avoidance hypothesis; Tanaka & Sung, 2016). As this study was conducted online, we were not able to collect information about looking patterns which would help us to tease apart these two competing hypotheses. Although we assume that our experimental manipulation led to increased looking to the eyes, stimuli were presented for 2000 ms, which could have given participants time to shift their gaze away from the eye region. Finally, we highlight that as in the CU analyses, when we adjusted for conduct problems the autistic traits*emotion interaction effect fell below the threshold for statistical significance.

Strengths of this paper include a moderately sized sample of well‐characterised youth with varying levels of CU and autistic traits, replication of previous results in an independent sample, and pre‐registered analyses. One key limitation, due to data collection being conducted online, is the lack of objective measurement of looking to the eyes. It is plausible that cueing to the eyes increased attention to the task more generally, rather than it is specific to the eyes. However, the specificity of effects for CU traits, with cueing associated with better recognition of fear, suggests that cueing to the eyes did indeed lead to increased time looking at the eye region; most information is held in the eyes in fearful expressions (Scheller et al., 2012). We also note that results, especially condition effects, could be in part driven by practice effects, as the uncued block was always presented before the cued block (so that exposure to cued trials did not bias performance in the uncued block). Future studies could consider randomly presenting uncued and cued trials within the same block and including order as a factor in statistical analyses in order to minimise practice effects. Additionally, we note that in the cued condition, parent education showed the opposite association to what we would predict – with lower emotion recognition for children of parents who had a degree or higher (although similar SES effects are seen elsewhere; Bedford et al., 2020). Given the generally high demographic of our families, it is difficult to interpret this unpredicted effect; future work is required across more representative and diverse samples.

Results have important implications, both in terms of methodology and clinical approach. With regards to study design, the differences between the uncued and cued conditions suggest that the location of attentional cues (e.g. fixation crosses) should be carefully considered in studies of facial emotion recognition and autistic and/or CU traits, as it may be an important source of heterogeneity in the existing literature. From a clinical perspective, results suggest that interventions which encourage children with CU traits to pay more attention to the eyes may be beneficial for emotion recognition (although preliminary evaluations have so far reported null effects; Dadds, English, Wimalaweera, Schollar‐Root, & Hawes, 2019), but similar interventions may be unhelpful for children with autistic characteristics. Results are especially relevant for the autism field, where many interventions, both explicitly and implicitly, rest on the assumption that social difficulties can be ameliorated by encouraging autistic children to make more eye contact.

## Supporting information


**Appendix S1.** Additional details of data collection.
**Appendix S2.** Additional details of data cleaning and processing.
**Table S1.** Full battery of experimental tasks.
**Table S2.** Emotion recognition task performance by diagnostic grouping.
