# Supplementary material for: Associations between emotion recognition and autistic and callous‐unemotional traits: differential effects of cueing to the eyes
Source: J Child Psychol Psychiatry. 2022 Dec 12;64(5):787–96. doi: 10.1111/jcpp.13736 (PMC10953425; doi:10.1111/jcpp.13736)
Supplement: Supplementary file 1 — Appendix S1. Additional details of data collection. Appendix S2. Additional details of data cleaning and processing. Table S1. Full battery of experimental tasks. Table S2. Emotion recognition task performance by diagnostic grouping. [file JCPP-64-787-s001.docx]

**Associations between Emotion Recognition and Autistic and Callous Unemotional Traits: Differential Effects of Cueing to the Eyes**

*Supporting Information*

***Appendix S1****. Additional Details of Data Collection*

Parent questionnaire data was collected online using Qualtrics and child cognitive task data was collected online using Gorilla on a laptop/computer using Google Chrome or Microsoft Edge. Parents were instructed to help their child set up the tasks (e.g., calibrating the size stimuli were presented at to their screen size), but then to leave the child to complete tasks alone as much as possible.

***Table S1.*** *Full Battery of Experimental Tasks*

| Task order | Experimental cognitive task |
| --- | --- |
| 1 | Uncued Emotion Recognition Task used to capture a measure of emotion recognition accuracy and looking to the eyes. |
| 2 | Visual Search Task used to capture a measure of exogenous and endogenous attentional control. |
| 3 | Spaceman Task used to capture a measure of inhibition/cognitive flexibility. |
| 4 | Cued Emotion Recognition Task used to capture a measure of emotion recognition accuracy using three conditions: no cue, nose cue and eye cue. |
| 5 | Social Information Processing Task used to capture a measure of hostile attribution bias. |
| 6 | Receptive One Word Picture Vocabulary Task – 4^th^ Edition (ROWVPT-4) used to capture a measure of proxy verbal IQ. |
| 7 | Frith-Happe Animations Task used to capture a measure of theory of mind intentionality and accuracy. |
| 8 | Choose A Movie (CAM) Task used to capture a measure of social motivation/preference. |
| 9 | Ambiguous Situations Task used to capture a measure of social and non-social interpretation biases. |

***Appendix S2.*** *Additional Details of Data Cleaning and Processing*

*Emotion Recognition Task*

At the trial level, trials with reaction times (RTs) below 200ms or 3 SDs above each participant’s mean task RT (2.5-2.7% of trials), or where Gorilla noted a stimulus loading delay (0.3% of trials) were excluded.

At the task level, data from the uncued and cued conditions were excluded if (a) the participant noted that the stimuli did not present properly (n = 0 uncued, n = 1 cued), (b) loading delays were present on ≥20% of trials (n = 0) (c) the stimulus did not render at the correct size (n = 1 uncued, n = 5 cued), or (d) participants had < 50% valid trials (n = 0 uncued, n = 1 cued). Following these exclusions, one participant was excluded, and the remaining participants had an average of 97% valid trials.

*Receptive One Word Picture Vocabulary Test – 4^th^ edition (ROWPVT-4)*

At the trial level, trials with RTs <200ms (0.2% of trials), or where stimuli had not properly loaded were excluded (< 0.1% of trials).

At the task level, data were excluded overall if the participant noted that the stimuli did not present properly on the task (n=2), and if loading delays were present on ≥ 20% of trials (n=0).

As a final check to ensure data quality, participants were presented with two attention checks throughout the whole task battery (e.g., shown six animal pictures and asked to click on the fish). All participants passed this criterion.

*Deviations from Pre-Registered Protocol*

We deviated from our pre-registered analysis in the following ways. Before running analyses, we reviewed descriptive statistics, and made the following decisions: 1) We recoded data where participants had <100% valid trials for an emotion (hence changing their possible scores from 0%, 25%, 50%, 75% or 100% to 33.33% or 66.66%), replacing total percentage accuracy scores of 33.33 (1.2% of trials) and 66.66 (2.2% of trials) to their nearest neighbouring category (25 and 75 respectively) to aid model convergence; 2) We had originally planned to include data from a letter-number grid task to capture spontaneous looking patterns when viewing emotional faces. Briefly, this involved presenting participants with a face stimulus for 500ms, followed by a grid of letters and numbers (e.g. A5, A6) for 500ms. Following the grid presentation, participants are given an open text box and are asked to type the clearest letter and number pair they saw. This measure is design to capture information about which part of the face participants look at when first presented with a face. However, based on the much higher rate of invalid or inconclusive responses on the letter-number grid task (8.5%) as compared to other tasks (0-0.5% in the emotion recognition task), and the fact that multiple participants reported not understanding the task (n=11), we did not further analyse data from this task, as we were not confident the task had robustly captured the domains of interest.

During analyses, we made the following decisions: 1) Instead of using both diagnostic group and autistic traits, we just used a measure of current autistic traits. This was because a significant proportion of children in the non-autistic group (n=16) met the suggested cut-off for autism spectrum on the SCQ-Lifetime (≥ 15), suggesting potential for diagnostic misclassification. Although we had originally planned to use the AQ-10, we instead used the recently developed SRS-Brief (Moul et al., 2015), as it was developed to improve the specificity of existing autistic trait measures in populations of children with co-occurring emotional and behavioural problems; 2) We had specified we would run models testing associations with autistic traits and CU traits separately; to maximise parsimony we decided to run models with both CU traits and autistic traits as simultaneous predictors. This allowed us to distinguish features associated with CU traits when accounting for autistic traits (and vice versa); and 3) We include all covariates of interest in our primary model instead of adding sequentially to minimise the number of analyses conducted.

|  |  | Mean (SD; range) | | | |
| --- | --- | --- | --- | --- | --- |
|  | Emotion | Non-Autistic, Low CU Traits (n=79) | Non-Autistic, High CU Traits (n=20) | Autistic, Low CU Traits (n=44) | Autistic, High CU Traits (n=28) |
| Uncued Condition | Anger | 87.97 (12.96; 25-100) | 83.75 (20.32; 25-100) | 76.14 (26.93; 0-100) | 86.61 (18.61; 50-100) |
|  | Happiness | 89.56 (15.30; 50-100) | 93.75 (11.11; 75-100) | 88.84 (17.43; 25-100) | 76.79 (29.60; 0-100) |
|  | Sadness | 90.51 (14.60; 50-100) | 92.50 (16.42; 50-100) | 83.52 (20.13; 25-100) | 85.71 (21.97; 25-100) |
|  | Fear | 59.49 (28.11; 0-100) | 51.25 (28.65; 0-100) | 48.30 (28.22; 0-100) | 41.96 (23.62; 0-75) |
|  | Surprise | 86.08 (19.91; 25-100) | 83.75 (23.33; 25-100) | 85.80 (20.46; 25-100) | 74.11 (17.32; 50-100) |
|  | Overall | 82.72 (9.77; 50-100) | 81.00 (12.31; 45-95) | 76.48 (13.96; 40-100) | 73.04 (12.12; 45-95) |
| Cued Condition | Anger | 94.81 (15.88; 25-100) | 86.25 (20.64; 50-100) | 81.98 (27.99; 0-100) | 92.59 (18.10; 25-100) |
|  | Happiness | 92.86 (17.61; 0-100) | 90.00 (17.01; 50-100) | 89.53 (17.45; 25-100) | 85.19 (21.10; 25-100) |
|  | Sadness | 93.51 (12.44; 50-100) | 92.50 (11.75; 75-100) | 91.86 (19.46; 0-100) | 79.63 (23.04; 25-100) |
|  | Fear | 58.12 (30.47; 0-100) | 65.00 (31.83; 0-100) | 52.33 (31.25; 0-100) | 64.81 (30.43; 0-100) |
|  | Surprise | 86.69 (19.27; 25-100) | 81.25 (26.75; 25-100) | 81.40 (22.55; 25-100) | 84.26 (20.97; 25-100) |
|  | Overall | 85.19 (12.15; 45-100) | 83.00 (13.02; 45-100) | 79.42 (14.81; 40-100) | 81.30 (12.53; 50-100) |

***Table S2.*** *Emotion Recognition Task Performance by Diagnostic Grouping*
